# Identification of a Novel Post-transcriptional Transactivator from the Equine Infectious Anemia Virus

**DOI:** 10.1128/jvi.01210-22

**Published:** 2022-11-30

**Authors:** Jiwei Li, Xiangmin Zhang, Bowen Bai, Mengmeng Zhang, Weiwei Ma, Yuezhi Lin, Xiaojun Wang, Xue-Feng Wang

**Affiliations:** a State Key Laboratory of Veterinary Biotechnology, Harbin Veterinary Research Institute of Chinese Academy of Agricultural Sciences, Harbin, China; Icahn School of Medicine at Mount Sinai

**Keywords:** Grev, Rev, equine infectious anemia virus, lentiviruses, post-transcriptional transactivator, transcript

## Abstract

All lentiviruses encode a post-transcriptional transactivator, Rev, which mediates the export of viral mRNA from the nucleus to the cytoplasm and which is required for viral gene expression and viral replication. In the current study, we demonstrate that equine infectious anemia virus (EIAV), an equine lentivirus, encodes a second post-transcriptional transactivator that we designate Grev. Grev is encoded by a novel transcript with a single splicing event that was identified using reverse transcription-PCR (RT-PCR) and RNA-seq in EIAV-infected horse tissues and cells. Grev is about 18 kDa in size, comprises the first 18 amino acids (aa) of Gag protein together with the last 82 aa of Rev, and was detected in EIAV-infected cells. Similar to Rev, Grev is localized to the nucleus, and both are able to mediate the expression of Mat (a recently identified viral protein of unknown function from EIAV), but Rev can mediate the expression of EIAV Gag/Pol, while Grev cannot. We also demonstrate that Grev, similar to Rev, specifically binds to rev-responsive element 2 (RRE-2, located in the first exon of mat mRNAs) to promote nuclear export of mat mRNA via the chromosome region maintenance 1 (CRM1) pathway. However, unlike Rev, whose function depends on its multimerization, we could not detect multimerization of Grev using coimmunoprecipitation (co-IP) or bimolecular fluorescence complementation (BiFC) assays. Together, these data suggest that EIAV encodes two post-transcriptional transactivators, Rev and Grev, with similar, but not identical, functions.

**IMPORTANCE** Nuclear export of viral transcripts is a crucial step for viral gene expression and viral replication in lentiviruses, and this export is regulated by a post-transcriptional transactivator, Rev, that is shared by all lentiviruses. Here, we report that the equine infectious anemia virus (EIAV) encodes a novel viral protein, Grev, and demonstrated that Grev, like Rev, mediates the expression of the viral protein Mat by binding to the first exon of mat mRNAs via the chromosome region maintenance 1 (CRM1) pathway. Grev is encoded by a single-spliced transcript containing two exons, whereas Rev is encoded by a multiple-spliced transcript containing four exons. Moreover, Rev is able to mediate EIAV Gag/Pol expression by binding to rev-responsive element (RRE) located within the Env-coding region, while Grev cannot. Therefore, the present study demonstrates that EIAV encodes two post-transcriptional regulators, Grev and Rev, suggesting that post-transcriptional regulation patterns in lentivirus are diverse and complex.

## INTRODUCTION

Lentiviruses, including HIV-1, are able to integrate their proviral DNA into the host genome and become an integral part of the host cell (1). Lentiviral transcription takes place in the nucleus, and the 5′ long terminal repeat (LTR) of the proviral DNA acts as a viral promoter to mediate the initiation of transcription by recruiting the RNA polymerase II of the host cell (2). A full-length lentiviral genomic RNA is first synthesized that encodes the viral structural protein Gag/Pol and is also packaged into nascent virions as genomic RNA. This primary transcript subsequently undergoes alternative splicing to generate multiple subgenomic spliced transcripts encoding other viral proteins (3). All of these transcripts, including fully spliced, incompletely spliced, and unspliced transcripts, utilize one of two distinct mechanisms for export from the nucleus to the cytoplasm for viral protein translation and virion assembly (3). In general, nuclear export of the fully spliced transcripts utilizes the endogenous cellular pathway used by cellular mRNA (4, 5), while nuclear export of incompletely spliced and unspliced transcripts utilizes the chromosome region maintenance 1 (CRM1)-dependent export pathway mediated by the virally regulatory protein Rev (6–8).

Rev is a regulatory protein shared by all lentiviruses. Rev acts as a post-transcriptional transactivator to promote the nuclear export of unspliced or incompletely spliced transcripts encoding viral structural proteins (Gag, Pol, and Env), by binding to a special RNA sequence (termed the Rev-responsive element [RRE]) located in the Env-coding region (9–12). Thus, Rev is required for expression of structural proteins and virus production (8). The molecular details of the Rev-mediated export of lentiviral mRNA have been well characterized, especially in HIV-1 (6, 13–17). A single Rev molecule binds to the RRE, begins assembly and recruits additional Rev molecules for multimerization in the nucleus, and then utilizes the CRM1 nuclear export pathway to transport the viral mRNA to the cytoplasm. Therefore, Rev nuclear localization, multimerization, binding to RRE, and interaction with the host protein CRM1 are essential for its role in mediating viral mRNA nuclear export (13, 14, 17–21).

Equine infectious anemia virus (EIAV) is a macrophage-tropic lentivirus that mainly infects Equus species. EIAV is currently believed to be the lentivirus with the genetically simplest genome (22). In addition to the three structural proteins (Gag, Pol, and Env) shared by lentiviruses, EIAV encodes two regulatory proteins (Tat and Rev) and one accessory protein (S2). In addition, our lab recently discovered a novel viral protein Mat in EIAV (23), but the biological function of the Mat is as yet unclear. The primate lentiviruses, such as human immunodeficiency virus (HIV) and simian immunodeficiency virus (SIV), encode two regulatory proteins (Tat and Rev) and four accessory proteins (Nef, Vpu, Vif, and Vpr/Vpx) (24). The regulatory proteins control lentiviral gene expression at the transcriptional (Tat) and post-transcriptional (Rev) levels (3), while the accessory proteins are involved in viral replication through interacting with host proteins (25, 26). EIAV Rev coding sequences contain two exons. The first Rev exon is in the same reading frame as the surface glycoprotein (SU) subunit of Env, and the second overlaps with the C-terminal coding region of the transmembrane protein (TM) subunit of Env but is in a different reading frame (27). Mutational analyses indicate that all functional domains of the EIAV Rev are located in the second exon, including the nuclear export signal (NES), the RNA-binding domain (RBD), and the nuclear localization signal (NLS) (28–31).

Previous research into EIAV transcription patterns has led to the discovery of five species of EIAV-specific mRNA transcripts, including full-length genomic RNA, a singly spliced transcript, and three fully spliced transcripts (32, 33). Rev is translated from a fully spliced bicistronic mRNA transcript, in which the other protein encoded is Tat (27). We recently discovered multiple novel transcripts in EIAV, one of which encodes Mat (23). Interestingly, the expression of Mat depends on the Rev/CRM1-mediated nuclear export pathway, and EIAV Rev could specifically bind to the first exon of mat mRNA to promote its nuclear export. Therefore, there must be two RREs in EIAV. The first RRE (RRE-1) is located in the Env-coding region and is responsible for the expression of viral structural proteins (34–36), and the second (RRE-2) overlaps with the first exon of mat mRNA located in the Gag-coding region and is responsible for the expression of Mat (23). In this study, we identified another novel transcript in EIAV containing a 303-bp open reading frame (ORF) consisting of two exons. The first exon (54 bp) overlaps with the N terminus of the *gag* gene, and the second (249 bp) overlaps with the C terminus of the *rev* gene, so the protein encoded by it was named Grev. The molecular weight of Grev is about 18 kDa, and it is mainly localized to the nucleus. Functionally, Grev is able to mediate Mat expression by binding RRE-2 via the CRM1 pathway but is not able to mediate Gag/Pol expression. In conclusion, we describe here a novel post-transcriptional transactivator from EIAV that updates our understanding of the organization of the EIAV genome and expands our knowledge of lentiviral post-transcriptional regulatory mechanisms.

## RESULTS

### Identification of a novel transcript from EIAV.

**FIG 1:**
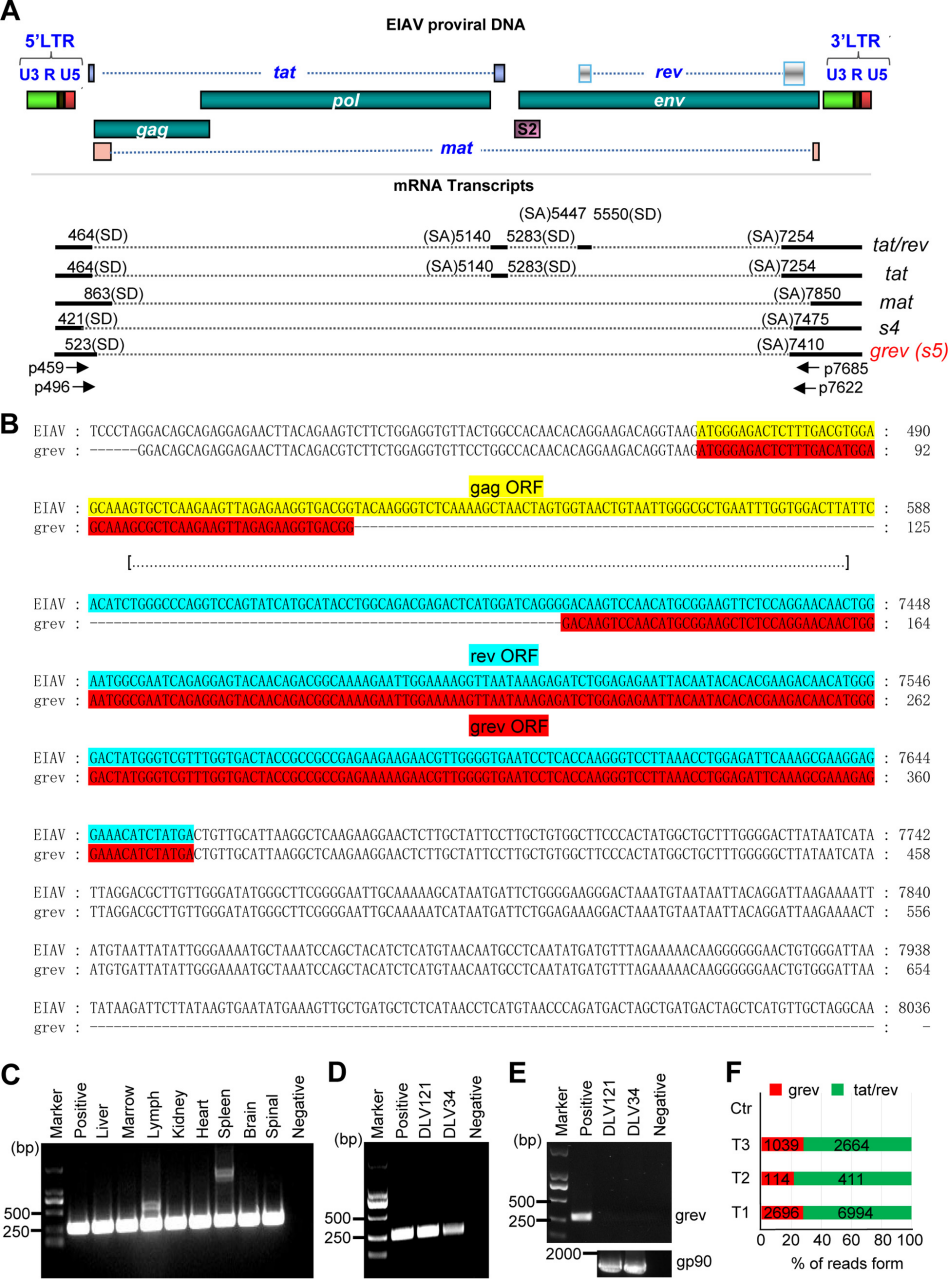
Identification of the grev transcript from equine infectious anemia virus (EIAV). (A) Organization of the EIAV proviral genome is displayed at the top of the figure. Known open reading frames (ORFs) are shown as rectangles with different colors. The splicing patterns of the five fully spliced transcripts from EIAV identified in previous studies are shown below the genomic structure. The numbers represent the splice sites of each transcript. LTR, long terminal repeat; SA, splice acceptor; SD, splice donor. Horizontal arrows show the locations of oligonucleotide primers for identification of the grev transcript. (B) Sequence alignment of the grev transcript and EIAV proviral genome (GenBank accession number GU385361.1). The predicted *grev*, *gag*, and *rev* ORFs are highlighted in red, yellow and blue, respectively. The intron in the processed grev transcript is omitted and is indicated by a dotted line. (C) Identification of grev transcript using mRNA extracted from the tissues of horses infected with EIAV_LN40_ using reverse transcription (RT)-PCR with the nested primer pairs p459/p7685 and p496/p7622, as indicated. PCR products were resolved on an agarose gel and visualized. (D, E) Identification of grev transcript in eMDMs infected with the EIAV strains DLV121 (EIAV_DLV121_) and DLV34 (EIAV_DLV34_). (D) Total RNAs extracted from equine monocyte-derived macrophages (eMDMs) infected with EIAV strains DLV121 and DLV34 were used as the templates for PCR amplification using the same method described in B. (E) Total cellular DNA extracted from eMDMs infected with EIAV_DLV121_ and EIAV_DLV34_ were used as the templates for PCR amplification with primers targeting *grev* (upper) and *gp90* (as a positive control for proviral DNA) (lower), respectively. (F) Identification of the grev transcript from eMDMs infected with EIAV using RNA-seq. The tat/rev transcript was used as the positive control. The vertical axis shows the number of reads with grev-specific (AATGATTGATG↑ATTGGGAAAA) and tat/rev-specific (TCTGTTATAAG↑CCATAAAGCA) splicing sites from three RNA-seq sequence libraries derived from eMDMs infected with EIAV_DLV121_. T1, T2, and T3 represent three independent cell samples infected with EIAV. Ctr, uninfected cell samples. The numbers in each rectangle indicate the number of reads with grev- or tat/rev-specific splicing sites in each sample.

In our previous study (23), multiple EIAV-specific transcripts were detected from EIAV-infected horse tissue by using the reverse transcriptase-PCR (RT-PCR) (Fig. 1A). For the present study, we focused on one of these transcripts, s5 (GenBank accession number ON994416). s5 is generated by single splicing event, with the 5′ splice donor site (SD) located at nucleotide (nt) 523 of the genomic sequence and the 3′ splice acceptor site (SA) located at nt 7410 of the genomic sequence (the reference sequence’s GenBank accession number is GU385361.1). This novel EIAV transcript includes a 303-bp open reading frame (ORF) consisting of two exons, with the first exon sharing the first 54 bp of the N terminus of the Gag-coding region, and the second overlapping the last 249 bp of the Rev coding region (Fig. 1B). Here, the s5 transcript will be referred to as the grev transcript, and its corresponding gene and protein are referred to as *grev* and Grev, respectively. Based on the splicing pattern of grev, We amplified partial (about 260 bp) grev transcript sequences by nested RT-PCR using mRNA extracted from various tissues of horses infected with EIAV_LN40_, including liver, testis, lymph nodes, kidney, heart, spleen, brain, and marrow, and obtained PCR bands of predicted sizes (Fig. 1C). The bands were purified, cloned, and sequenced to confirm that they contained the s5-specific splicing sites SD^523^ and SA^7410^. The s5-specific bands were also amplified using mRNA extracted from the equine monocyte-derived macrophages (eMDMs) infected with EIAV_DLV121_ and EIAV_DLV34_ *in vitro* (Fig. 1D) but could not be amplified using genomic DNA extracted from these cells (Fig. 1E), suggesting that grev transcript is derived from EIAV-specific mRNA and not EIAV proviral DNA. In addition, the abundance of viral mRNA arising from the EIAV genome was analyzed based on our previously published RNA-seq data from eMDMs infected with EIAV_DLV121_ (37). Using reads with tat/rev-specific splicing sites as controls, we found reads with *grev*-specific splicing sites in the eMDMs infected with EIAV, but not in the uninfected eMDMs (Fig. 1F). These results indicated that the grev transcript is also present in EIAV-infected tissue *in vivo* and cells *in vitro*.

### Expression of Grev in EIAV-infected cells.

**FIG 2:**
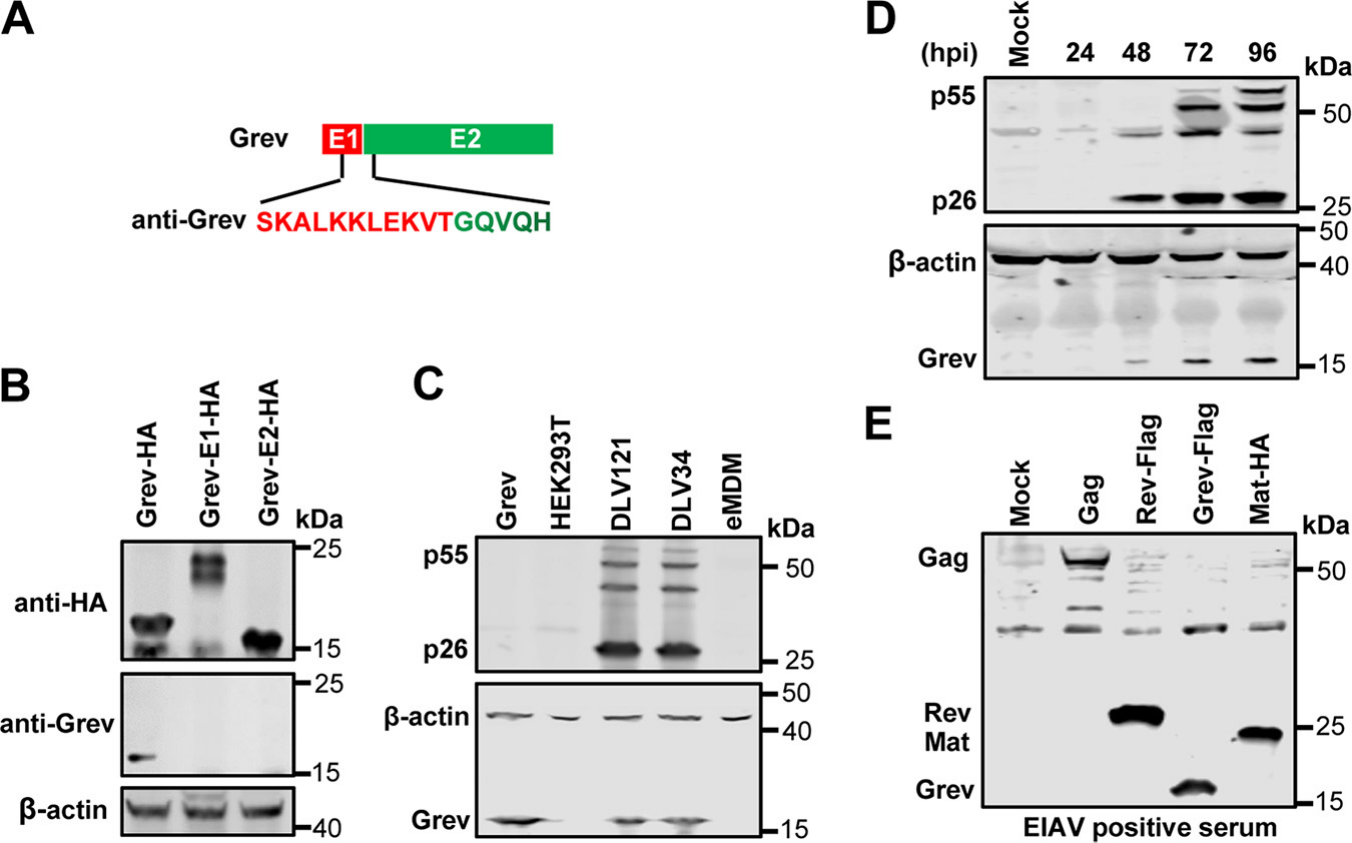
Expression of Grev in EIAV-infected cells. (A) Schematic diagram of the antigenic epitope for the preparation of anti-Grev antibody. E1, the first exon; E2, the second exon. (B) Identification of the specificity of the anti-Grev monoclonal antibody. The full-length Grev (Grev-hemagglutinin [HA]) expression plasmid, the expression plasmid that express first 131 amino acid residues of Gag, which include the first exon of Grev (Grev-E1-HA), and the plasmid expressing the second exon of Grev (Grev-E2-HA) were transfected individually into HEK293T cells. At 48 h post-transfection (hpt), the expression levels of protein were assessed with Western blotting (WB) using an anti-HA monoclonal antibody and an anti-Grev monoclonal antibody. (C) Detection of Grev expression in equine monocyte-derived macrophages (eMDMs) infected with EIAV. The eMDMs were infected with EIAV_DLV34_ or EIAV_DLV121_. Cells were collected at 96 h postinfection (hpi) and were lysed for the detection of Gag and Grev expression using anti-p26 antibody and anti-Grev antibody. Uninfected eMDM was used as a negative control, and HEK293T cells transfected with Grev were used as a positive control. (D) Time course analyses of Grev expression. The eMDM cells were infected with EIAV_DLV121_ at a multiplicity of infection (MOI) of 5. At each indicated time point, the total cell lysates were analyzed with WB with an anti-p26 monoclonal antibodies, an anti-Grev antibody, and an anti-action antibody. (E) Reactivity of EIAV-infected horse serum with grev by WB assay. Grev and other EIAV protein (Gag, Rev, and Mat) expression plasmids (VR-Grev-FLAG, VR-Gag, and VR-Rev-FLAG pcDNA-optMat-HA) were transfected individually into HEK293T cells. At 48 hpt, WB analysis of the cell lysates was performed using EIAV-positive horse serum. All of the experiments were performed three times, and a representative result is shown.

To investigate whether the Grev protein is expressed in EIAV-infected cells, we prepared mouse-derived monoclonal antibodies (MAbs) against synthetic peptides (SKALKKLEKVTGQVQH) corresponding to the translation products at the junction of the first and second exons of Grev (Fig. 2A) and confirmed that this MAb could only recognize full Grev, but not the epitope corresponding to the first or second exon (Fig. 2B), which implies that the antibody does not recognize the corresponding parts of the matrix protein (MA) or Rev. Using this antibody, a protein of about 18 kDa was detected in eMDMs infected with either EIAV_DLV121_ or EIAV_DLV34_ and in HEK293T cells transfected with the Grev expression plasmid (as a positive control) using Western blotting (WB), whereas no protein was detected in uninfected eMDMs or untransfected HEK293T cells (Fig. 2C). We also observed that Grev expression increased gradually with time after infection of eMDM cells with EIAV (Fig. 2D). In addition, we confirmed that EIAV-positive horse serum recognizes Grev (Fig. 2E). These results indicated that the Grev protein is expressed in EIAV-infected eMDM cells.

### EIAV Grev promotes export and expression of mat mRNA.

**FIG 3:**
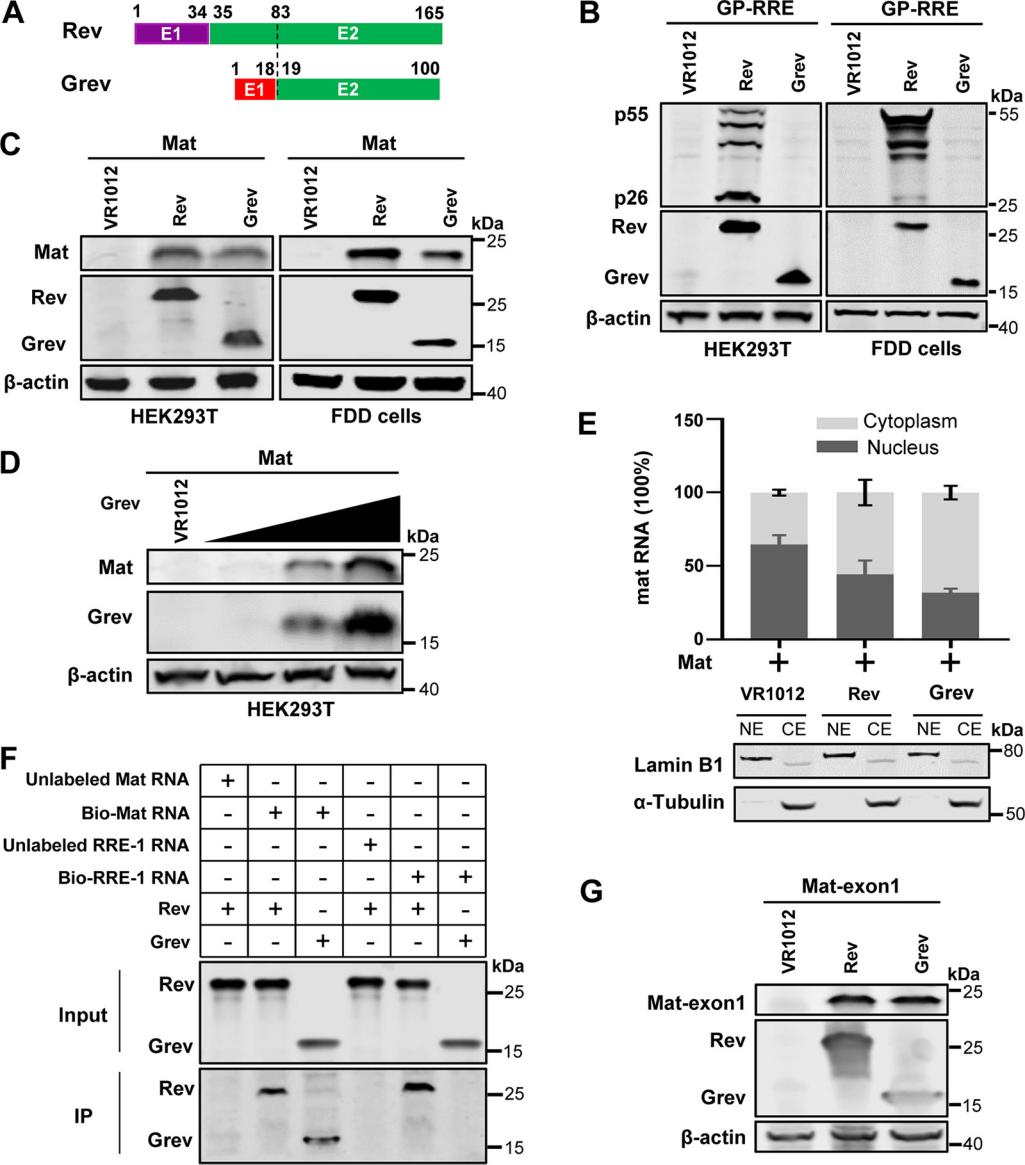
Grev promotes the expression of Mat. (A) Schematic diagram of the Rev and Grev expression constructs. E1, the first exon; E2, the second exon. (B) Grev could not promote expression of EIAV Gag/pol. HEK293T cells (left) and fetal donkey dermal (FDD) cells (right) were cotransfected with plasmids expressing Gag/Pol (GP-RRE), FLAG-tagged Rev, or Grev. The cells were collected at 48 hpt and were lysed for the detection of Gag (p55), Rev, and Grev expression using anti-p26 antibody and anti-FLAG antibody. (C) Grev promotes the expression of EIAV Mat. HEK293T cells (left) and FDD cells (right) were cotransfected with plasmids expressing HA-tagged Mat and FLAG-tagged Rev or Grev. Cells were collected at 48 hpt and lysed. Expression of Mat, Rev, and Grev was assessed using anti-HA antibody and anti-FLAG antibody. (D) A Mat expression plasmid was transfected into HEK293T cells along with increasing amounts of Grev expression vector (0, 0.5, 1, and 2 μg). The expression levels of Mat and Grev were detected at 48 hpt with WB using anti-HA antibody and anti-FLAG antibody. (E) RT-quantitative PCR (qPCR) analysis was performed to determine distribution of the mat mRNAs in the nucleus and the cytoplasm (upper). HEK293T cells were cotransfected with Mat expression vector and Grev or Rev expression plasmids. RNA samples from the nuclear and cytoplasmic fractions were extracted at 36 hpt, and then qPCR analysis was performed to quantify the samples absolutely using standard curves. The ratio of nuclear and cytoplasmic mRNAs from the same sample was then analyzed. Error bars represent standard deviations from three independent experiments. LaminB1 and α-tubulin were detected using WB to assess the efficacy of cell fractionation (lower). NE, nuclear extracts; CE, cytoplasmic extracts. All of the experiments were performed three times, and a representative result is shown. (F) An RNA pulldown assay was performed to verify the interaction of mat mRNA with Grev protein. The mat mRNAs were synthesized by *in vitro* transcription, labeled with biotin, and then incubated with HEK293T cell lysate transfected with either Grev or Rev. The lysate was then incubated with streptavidin (STA) beads, after which the copurified proteins were subjected to WB using anti-FLAG antibody. The Rev pulled down by mat mRNA was used as a positive control. Non-biotin-labeled mat mRNAs and RRE-1 mRNAs were used as specificity controls. (G) Grev promotes the expression of the first exon of Mat (Mat-exon1). HEK293T cells were cotransfected Mat-exon1 expression constructs with Grev or Rev expression plasmids. At 48 hpt, the cells were harvested and subjected to WB. Mat-exon1 expression was assessed using anti-HA antibody, and Rev or Grev expression was assessed using anti-FLAG antibody. IP, immunoprecipitation.

Recently, we confirmed the existence of two independent Rev-responsive elements (RRE) in EIAV, namely, RRE-1, located in the Env-coding region, and RRE-2, located in the Gag-coding region (23). Rev binds to RRE-1 to mediate the expression of Gag/Pol and to RRE-2 to mediate the expression of the viral protein Mat. Considering that the major part of the Grev sequence overlaps with the C-terminal region of Rev (Fig. 3A), which contains several important functional domains of Rev (38), we investigated whether Grev has a post-transcriptional transactivator function similar to that of Rev. First, a Rev-dependent EIAV Gag/Pol expression vector with RRE-1 (pGP-RRE) was transfected into HEK293T cells or fetal donkey dermal (FDD) cells (a permissive cell for EIAV) in the presence of Rev or Grev expression plasmids. The amounts of Gag/Pol in the cell lysate were measured as the levels of Gag protein (p55) using WB. We found that Gag was detected in the presence of Rev, but not in the presence of Grev (Fig. 3B). Next, a Mat construct was transfected into HEK293T cells or FDD cells in the presence of Rev or Grev expression plasmids. We found that both Rev and Grev could mediate the expression of Mat (Fig. 3C), and Grev-mediated Mat expression was dose-dependent in HEK293T cells (Fig. 3D). These results suggest that Grev is able to mediate the expression only of Mat, unlike Rev, which can mediate the expression of both Mat and Gag/Pol.

To examine whether Grev, like Rev, promotes the export of mat mRNAs from the nucleus to the cytoplasm, we isolated nuclear and cytoplasmic mRNAs from HEK293T cells after cotransfection with Mat and Grev or Rev, and the mat mRNAs were analyzed using quantitative PCR (qPCR). We observed that most of the mat mRNAs were retained in the nucleus when the Mat construct was transfected alone, whereas the proportion of mat mRNAs in the cytoplasm increased markedly in the presence of Grev or Rev (Fig. 3E). To further assess whether Grev, like Rev, interacts with mat mRNAs, an RNA pulldown assay was performed using biotin-labeled mat mRNA and streptavidin (STA) beads, with RRE-1 mRNA as a specificity control. WB analysis with anti-FLAG antibody demonstrated that mat mRNA can bind to both Rev and Grev, while RRE-1 is able to bind only Rev but not Grev (Fig. 3F). In addition, we also confirmed that Grev can, like Rev, mediate the expression of the first exon of Mat (Mat-exon1), which overlaps with RRE-2 (Fig. 3G). These results show that Grev does not bind RRE-1 as Rev does. However, Grev, similarly to Rev, is able to promote the nuclear export of mat mRNA through specifically binding to RRE-2. Taken together, these data demonstrate that Grev, like Rev, can bind to RRE-2 to mediate the expression of Mat, but cannot bind to RRE-1 and therefore cannot mediate the expression of Gag/Pol.

### Identification of key functional domains on Grev important for the mediation of Mat expression.

**FIG 4:**
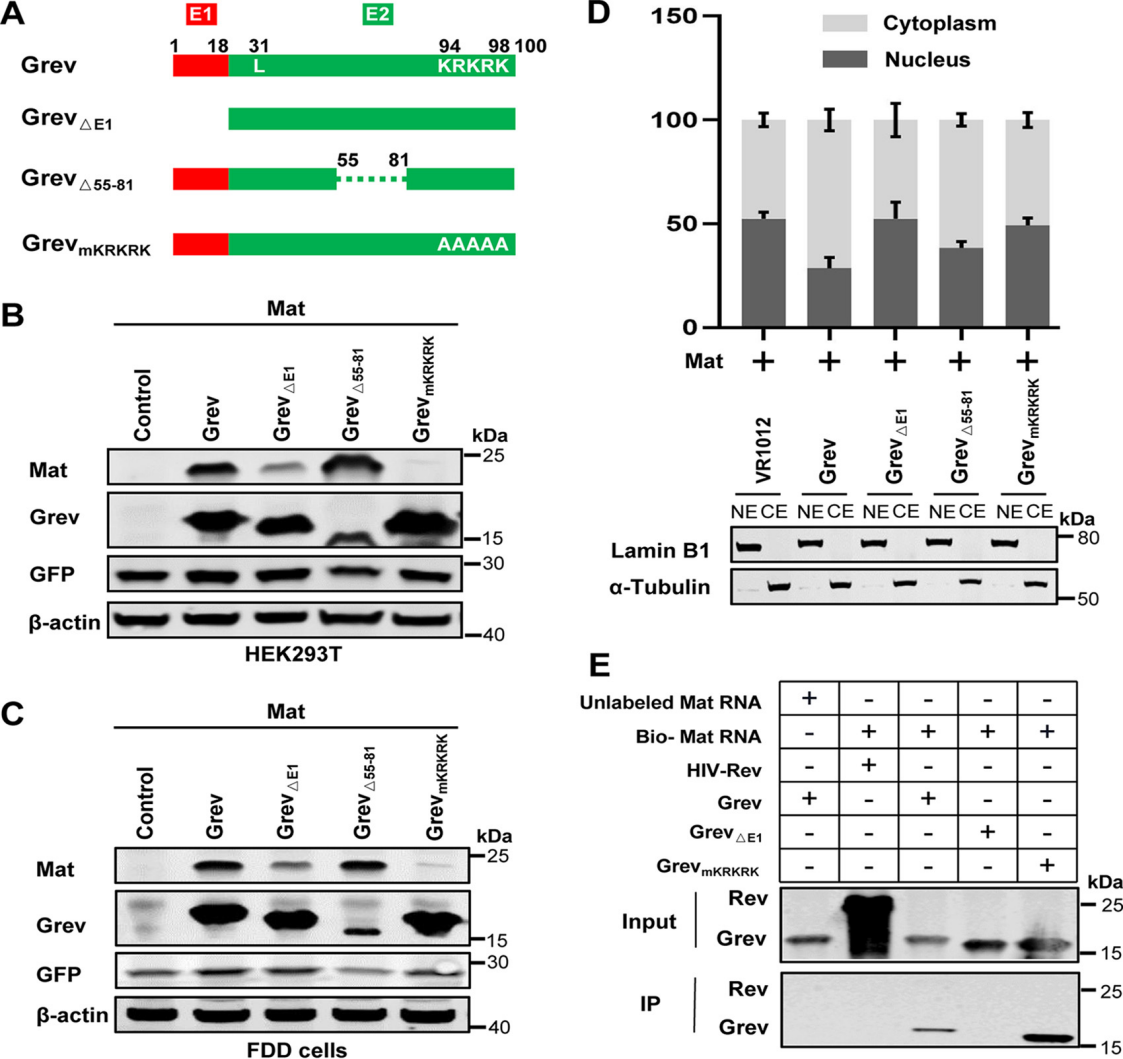
Identification of Grev key functional domains important for the mediation of Mat expression. (A) Schematic diagram of wild-type Grev (Grev) and the structures of the respective mutants. (B, C) HEK293T cells (B) or FDD cells (C) were cotransfected with Mat expression vector and a panel of plasmids expressing Grev or a mutant construct, as indicated. At 48 hpt, the cells were lysed, and the Mat and Grev proteins in the transfected cells were analyzed with WB. GFP, derived from pEGFP-N1, was quantified using a mouse anti-GFP antibody to monitor transfection efficiencies. This experiment was performed three times, and a representative result is shown. (D) Distribution of cytoplasmic and nuclear mat mRNAs in HEK293T cells at 36 hpt was analyzed using RT-qPCR. For these experiments, a Mat expression plasmid was transfected into HEK293T cells together with one of a series of Grev expression plasmids. RNA extraction and analysis were performed as in Fig. 3E. Error bars represent the standard deviations of three independent experiments. All of the experiments were performed three times, and a representative result is shown. LaminB1 and α-tubulin were detected using WB to assess the efficacy of cell fractionation. NE, nuclear extracts; CE, cytoplasmic extracts. (E) An RNA pulldown assay was performed to assay the interaction of mat mRNA with the various Grev mutants. The HIV-Rev pulled down by mat mRNA acted as a specificity control. Non-biotin-labeled mat mRNAs were also used as specificity controls.

We have shown that Grev, like Rev, specifically binds to mat mRNA and promotes the expression of Mat. To identify crucial functional domains of Grev, we constructed a series of Grev mutants (Fig. 4A): Grev_ΔE1_ lacked the first exon of Grev; Grev_Δ55-81_ contained a 27-amino-acid deletion (located in aa 55 to 81 of Grev) corresponding to the amino acids required for Rev-mediated Mat expression (23); and Grev_mKRKRK_, where the ^94^KRKRK^98^ of C-terminal of Grev is replaced with five alanines, which, corresponding to the all basic amino acid KRRRK, is a nuclear localization signal (NLS) and the RNA-binding motif of Rev (30). HEK293T cells were cotransfected with a Mat construct and an expression vector carrying Grev or one of its mutants, and levels of Mat protein in the cells were then analyzed using WB to test the nuclear export activity of each Grev mutant. Expression levels of Mat were significantly reduced in the presence of Grev_ΔE1_ or Grev_mKRKRK_ compared to those in the presence of wild-type Grev (Grev), but Mat expression levels did not change in the presence of Grev_Δ55-81_ (Fig. 4B). However, the mutants Grev_ΔE1_ and Grev_mKRKRK_ were expressed at levels equal to or higher than Grev, while Grev_Δ55-81_ expression levels were markedly lower than those of Grev (Fig. 4B). A similar result was observed in FDD cells (Fig. 4C). These results indicated that the first exon and the ^94^KRKRK^98^ motif of Grev are important for Grev-mediated Mat expression.

Furthermore, qPCR assay results also indicated that mat mRNAs were mainly retained in the nucleus in the presence of Grev_△E1_ or Grev_mKRKRK_, which is similar to the results obtained in the absence of Grev, while the largest proportion of mat mRNAs was found in the cytoplasm in the presence of either Grev or Grev_△55-81_ (Fig. 4D). Subsequently, to assess whether mat mRNAs interact with any of the Grev mutants, an RNA pulldown assay was performed using biotin-labeled mat mRNAs. WB analysis showed that mat mRNA was able to bind to Grev and Grev_mKRKRK_, but not to Grev_△E1_ or HIV Rev (the specific control) (Fig. 4E). These data indicate that the first exon of Grev is required for its binding to mat mRNA.

### Grev-mediated Mat expression is dependent on the CRM1 export pathway.

**FIG 5:**
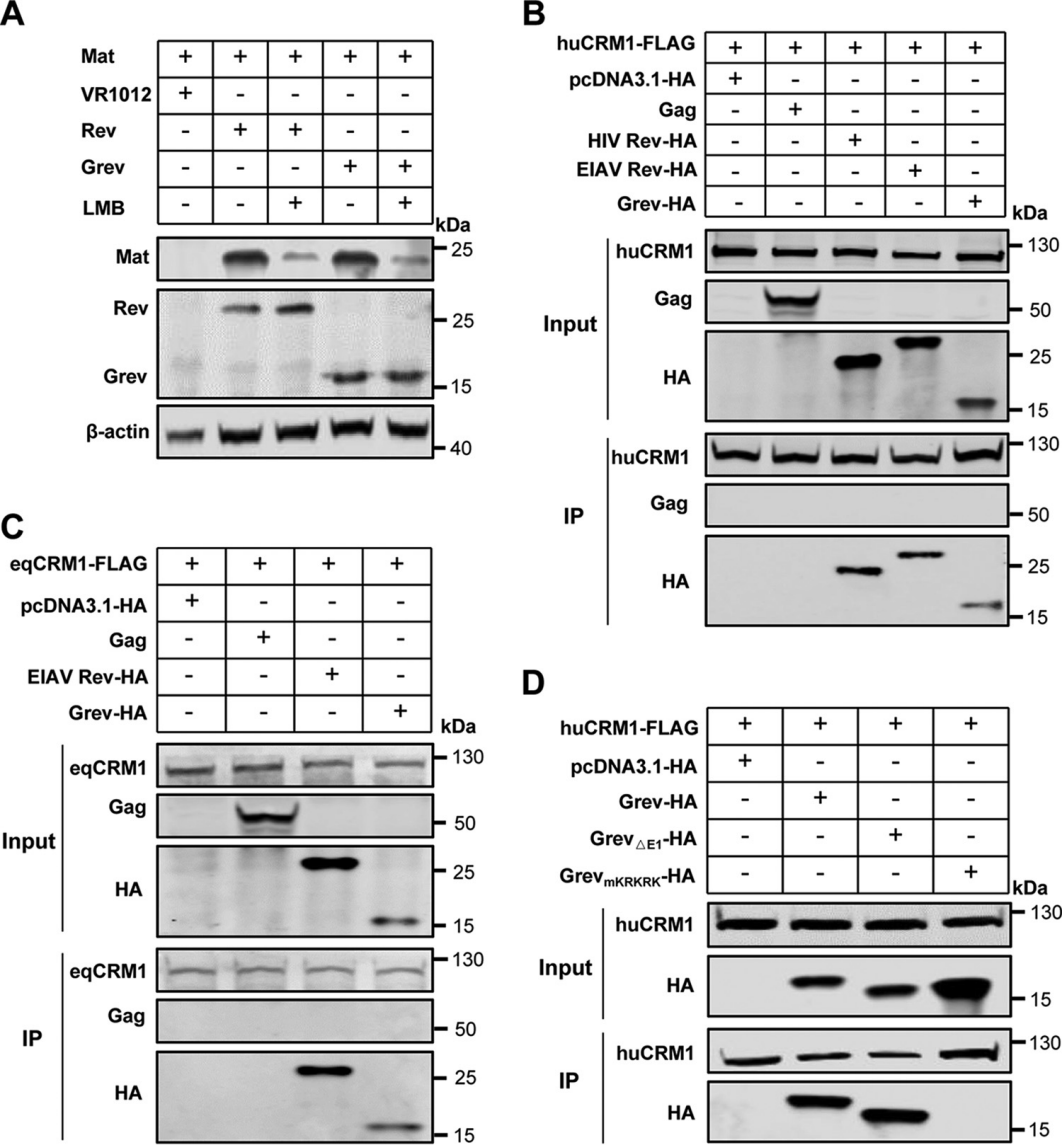
Interaction of Grev with chromosome region maintenance 1 (CRM1). (A) Effects of leptomycin B (LMB) on Grev-mediated Mat expression. HEK293T cells were cotransfected with plasmids expressing HA-tagged Mat, FLAG-tagged Rev, or Grev. LMB (50 nM) or dimethyl sulfoxide (DMSO) was added at 40 hpt, and the samples were incubated for a further 8 h. Cells were collected at 48 hpt and were lysed for the detection of Mat, Rev, and Grev expression using anti-HA antibody and anti-FLAG antibody. (B, C) Interaction of Grev and CRM1. HEK293T cells were cotransfected with plasmids carrying HA-tagged Grev and either FLAG-tagged huCRM1 (B) or eqCRM1 (C), as indicated. At 48 hpt, whole-cell lysates were immunoprecipitated using FLAG beads and then analyzed with WB. FLAG- and HA-tagged HIV Rev or EIAV Rev were used as positive controls, and Gag was used as a negative control. (D) CRM1-binding activity of Grev mutants (Grev_ΔE1_ and Grev_mKRKRK_) as described for panels B and C.

Considering that lentiviral Rev-mediated viral mRNA nuclear export is dependent on the CMR1 pathway (20, 39), we next tested whether Grev-mediated Mat expression was also dependent on the CRM1 export pathway. HEK293T cells were cotransfected with Mat and either Grev or Rev expression plasmids and were then treated with leptomycin B (LMB), which is an inhibitor of CRM1 and which blocks CRM1-mediated export from the nucleus. As expected, LMB was able to inhibit Rev- or Grev-mediated Mat expression and decrease the levels of Mat (Fig. 5A). To provide further evidence that the Grev-mediated Mat expression was dependent on the CRM1 pathway, we performed a coimmunoprecipitation (co-IP) assay to test the interaction between Grev and CRM1 in HEK293T cells. First, we co-expressed FLAG-tagged human CRM1 (huCRM1) together with either hemagglutinin (HA)-tagged Grev or EIAV Rev in HEK293T cells, and then we assessed the binding between huCRM1 and Grev or Rev using FLAG antibody-based co-IP. In order to rule out nonspecific binding, we employed HIV-1 Rev as a positive control and EIAV Gag as a negative control. The results showed that both Grev and Rev have a direct interaction with HuCRM1 (Fig. 5B). We used the same method to demonstrate that both Grev and Rev can also bind to equine CRM1 (eqCRM1) (Fig. 5C). Together, we conclude that Grev, like Rev, mediates Mat expression in a CMR1-dependent manner.

The above study showed that deletion of the first exon of Grev or mutation of the ^94^KRKRK^98^ motif attenuated or inactivated its activity in the mediation of Mat expression (Fig. 4), so we examined whether these two regions are critical for Grev binding to CRM1. A co-IP assay was performed to check the interaction between CRM1 and Grev_ΔE1_ or Grev_mKRKRK_ in HEK293T cells, with wild-type Grev used as a positive control. As shown in Fig. 5D, huCRM1 was able to coimmunoprecipitate Grev_ΔE1_, but not Grev_mKRKRK_. These data indicate that the ^94^KRKRK^98^ motif of Grev is essential for its binding to CRM1.

### Grev cannot form multimers.

**FIG 6:**
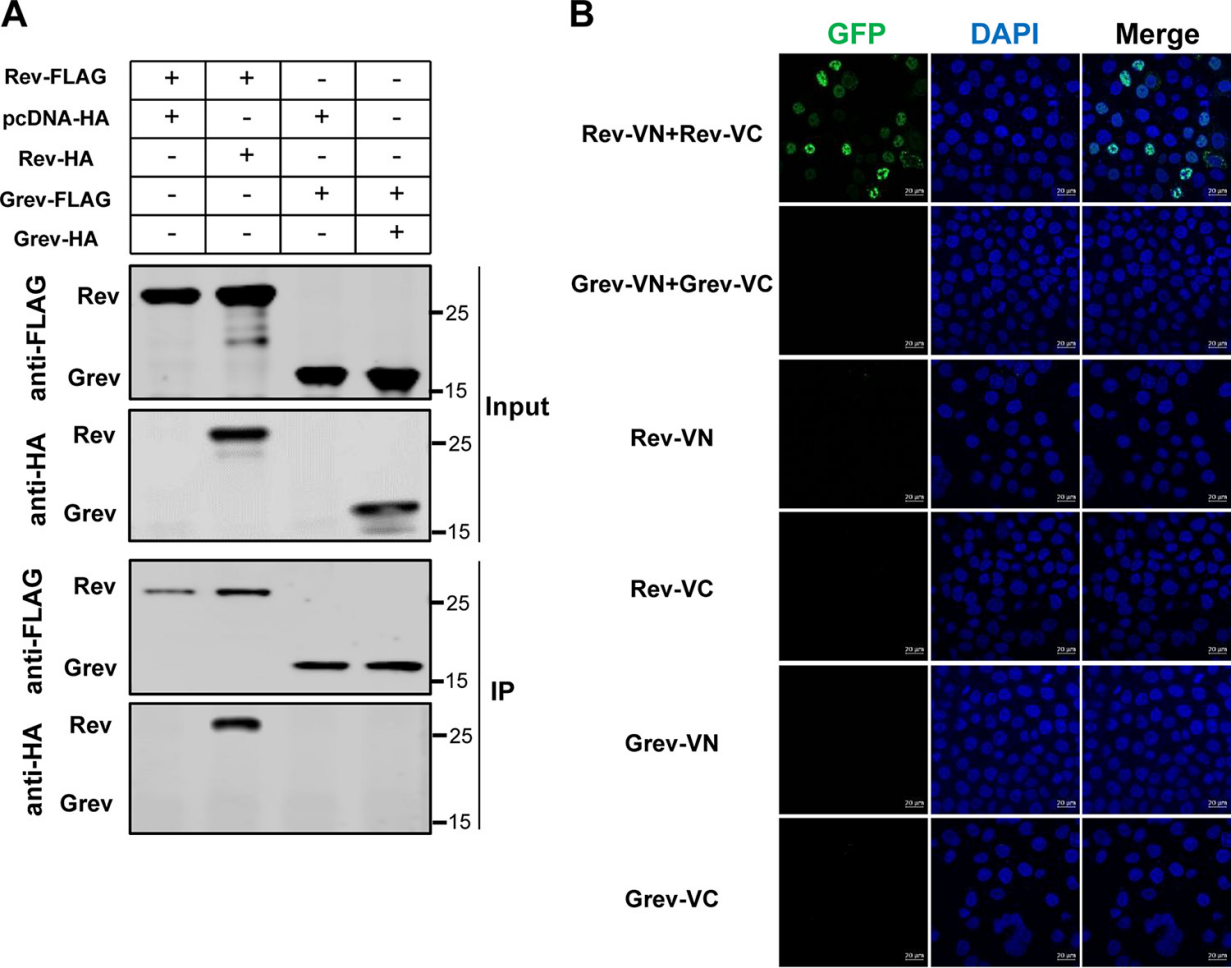
Grev-Grev interactions within cells. (A) Coimmunoprecipitation experiment to investigate Grev-Grev interactions. HEK293T cells were transfected with plasmids carrying FLAG- and HA-tagged Grev either alone or in combination as indicated. At 48 hpt, whole-cell lysates were immunoprecipitated using FLAG beads and then analyzed with WB. FLAG- and HA-tagged Rev were used as positive controls. (B) Bimolecular fluorescence complementation (BiFC) experiment to investigate Grev-Grev interactions. HeLa cells were transfected with the indicated plasmids. At 48 hpt, the cells were fixed, and fluorescent signals were visualized using confocal microscopy. Rev was used as a positive control. The green spots represent interacting complexes of the examined proteins. Bar, 20 μm. DAPI, 4′,6-diamidino-2-phenylindole; GFP, green fluorescent protein. VC, C-terminal 154 to 238 amino acids (aa) of a green fluorescent protein Venus; VN, N-terminal 2 to 173 aa of a green fluorescent protein Venus.

Previous studies have shown that the lentiviral Rev exists in a multimeric form and that this multimerization is required for the nuclear export activity of Rev (39, 40). To investigate whether Grev exists also in a multimeric form in cells, FLAG- and HA-tagged Grev were coexpressed in HEK293T cells, and Grev self-association was assessed using co-IP, with FLAG- and HA-tagged Rev used as the positive control. The results demonstrated that although Rev forms self-associations, Grev does not (Fig. 6A). We tested the self-association of Grev further using a bimolecular fluorescence complementation (BiFC) assay that directly visualizes protein-protein interactions in cells. The C terminus of Grev was fused either to the N-terminal 2 to 173 aa of a green fluorescent protein Venus (VN) or to its C-terminal 154 to 238 aa (VC). As a control, the Rev C terminus was also fused to either VN or VC. The Grev-VN and Grev-VC fusion proteins were then coexpressed in HeLa cells. No specific BiFC fluorescence signal was observed in cells cotransfected with Grev-VN and Grev-VC, whereas a strongly specific fluorescence signal was detected in those cotransfected with Rev-VN and Rev-VC and was virtually present in the nucleus (Fig. 6B). As a negative control, each of the fusion proteins was independently coexpressed with the complementary empty vector in HeLa cells. No fluorescence signal was detected in the negative controls (Fig. 6B). These data suggested that Grev may not exist in a multimeric form in cells.

### Grev is localized to the nucleus.

**FIG 7:**
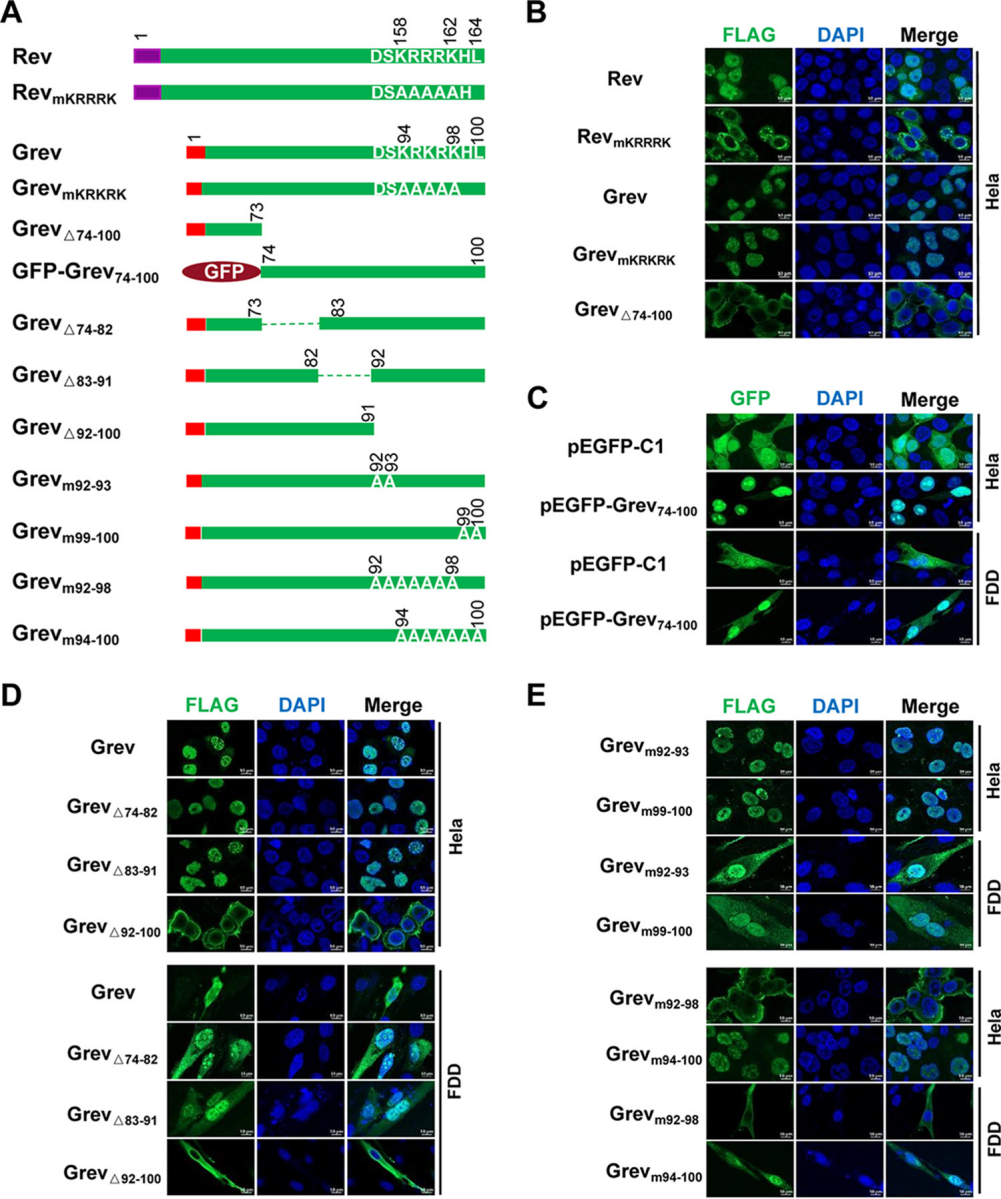
Identification of the nuclear localization signal (NLS) in Grev. (A) Schematic diagram of a series of plasmids used for the identification of the NLS in Grev. EIAV Rev and its mutant were used as the controls. The numbers represent the amino acid positions. The purple rectangle identifies the amino acid sequence encoded by the first exon of Rev, and the red rectangle represents that of the first exon of Grev. The green rectangle represents the amino acid sequence encoded by the second exon of Grev or Rev. All plasmids were transfected into HeLa or FDD cells individually, and the subcellular localization of each protein was examined using confocal microscopy 48 hpt. (B) Grev is localized to the nucleus. HeLa cells were transfected with the indicated plasmids. At 48 hpt, the cells were fixed and stained with an anti-FLAG antibody (green), the nuclei were stained with DAPI (blue), and expression was analyzed using confocal microscopy. (C) Amino acids 74 to 100 of Grev possibly contain an NLS. HeLa (upper) and FDD (lower) cells were transfected with either GFP or GFP-Rev_74-100_ expression plasmids. At 48 hpt, the cells were fixed, the nuclei were stained with DAPI (blue), and fluorescent signals were visualized using confocal microscopy. (D) Amino acids 92 to 100 of Grev are crucial for its nuclear localization. HeLa (upper) and FDD (lower) cells were transfected with Rev_△74-82_, Rev_△83-91_, and Rev_△93-100_ expression plasmids, individually. The Grev expression plasmid was used as a control. (E) The DSKRRRK motif in the C terminus of Grev is required for its nuclear localization. HeLa and FDD cells were transfected with Rev_m92-93_, Rev_m99-100_, Rev_m92-98_, and Rev_m94-100_ expression plasmids, individually. Immunofluorescence analysis was subsequently performed as in panel B. Bar, 20 μm.

Given that Rev is localized in the nucleus and that the KRRRK motif at the C-terminal of Rev was identified as a NLS (30, 31), we tested the whether Grev and Rev share similar subcellular localization. As shown in Fig. 7A, a series of Grev expression plasmids was constructed, with Rev and its mutant expression plasmids used as controls. Plasmids were transfected individually into HeLa or FDD cells, and subcellular localization of each protein was assessed using confocal microscopy. We observed that wild-type Grev (Grev) and wild-type Rev (Rev) largely localized to the nucleus. Grev_mKRKRK_, which contains alanine substitutions of the Grev ^94^KRKRK^98^ motif, was observed in the nucleus, while Rev_mKRRRK_, which contains alanine substitutions of the ^158^KRRRK^162^ motif of Rev, was observed only in the cytoplasm (Fig. 7B). These results suggest that Grev is located predominantly in the nucleus and that its nuclear localization is not dependent on the C-terminal basic amino acid motif ^94^KRKRK^98^. Next, we used an NLS predictor program (https://www.novopro.cn/tools/nls-signal-prediction.html) to analyze the amino acid sequence of Grev and identified a potential NLS located between aa 74 and 100. To verify this result, we deleted 27 amino acids (aa 74 to 100) from Grev and observed that the mutant, Grev_△74-100_, was localized to the cytoplasm. In addition, when these 27 aa were inserted into the C terminus of green fluorescent protein (GFP), the fusion protein GFP-Grev_74-100_ was found only in the nucleus, while GFP was distributed throughout the cytoplasm and nucleus (Fig. 7C). This result suggests that amino acids 74 to 100 are crucial for the nuclear localization of Grev and possibly contain an NLS.

To identify key regions in Grev important for nuclear localization, we constructed three deletion mutants, in which segments of the amino acids 74 to 100 (one-third each) were deleted (Grev_△74-82_, Grev_△83-91_, and Grev_△92-100_) and found that only Grev_△92-100_ localized to the cytoplasm, while the other two mutants localized to the nucleus (Fig. 7D). This suggests that the NLS of Grev is located between amino acids 92 and 100. To further investigate the specific position of the NLS in Grev, four Grev mutants with combinational alanine substitutions were constructed. From the experiments described above, it is clear that alanine substitutions in the ^94^KRKRK^98^ motif do not affect the nuclear localization of Grev (Fig. 7B). Moreover, as shown in Fig. 7E, combinational alanine substitutions of amino acids 92 to 93 or amino acids 99 to 100 of Grev also did not alter its nuclear localization. However, combinational mutation of amino acids 92 to 98 in Grev abrogated its nuclear localization, while mutation of amino acids 94 to 100 did not (Fig. 7E). Therefore, these results suggest that the NLS of Grev may be DSKRKRK rather than the basic motif KRKRK.

## DISCUSSION

**FIG 8:**
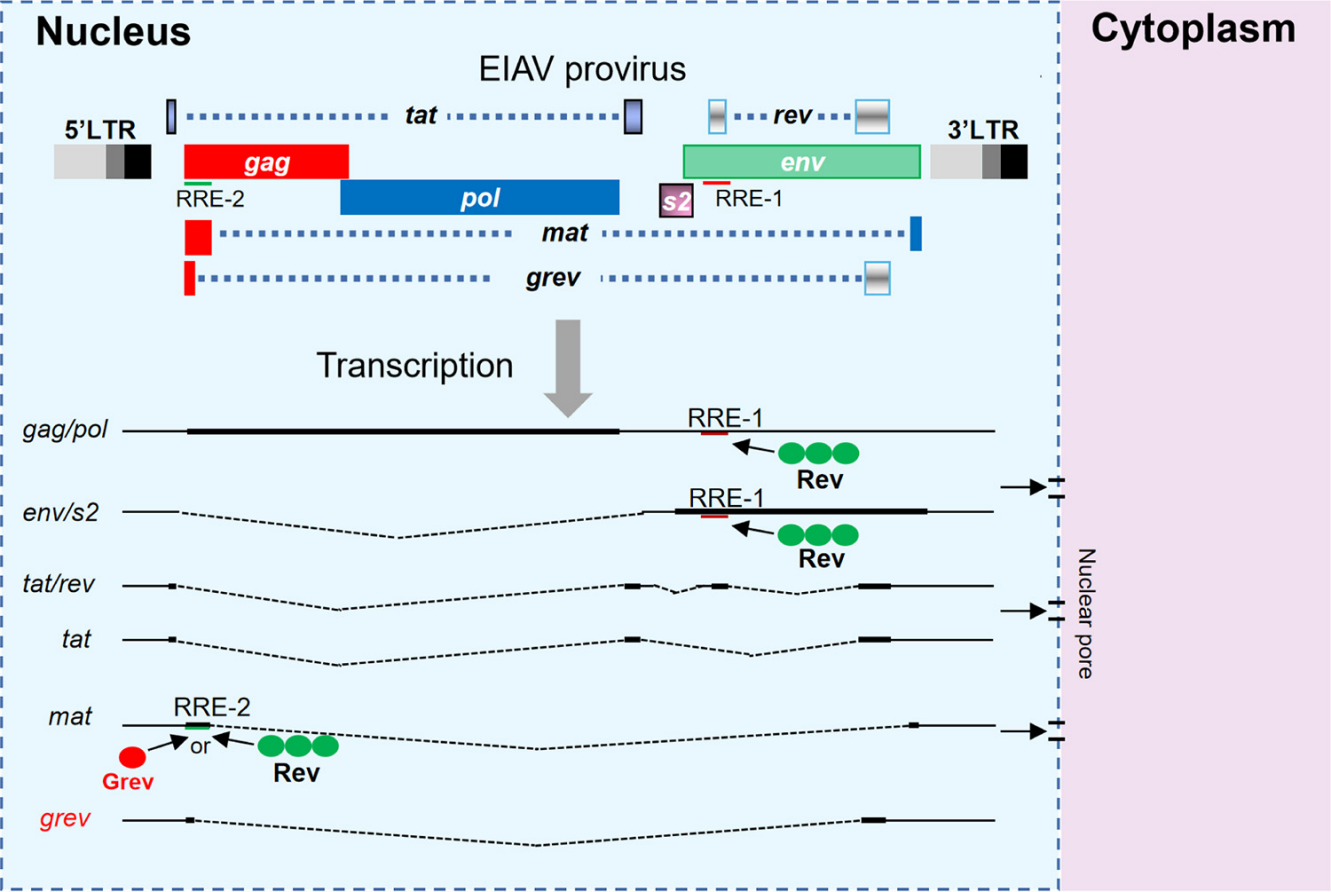
Structure of EIAV proviral genome and transcriptional pattern. A schematic diagram of the EIAV proviral genome is displayed at the top of the figure. The colored rectangles indicate open reading frames (ORFs) and are labeled with the gene names. Long terminal repeats (LTRs) are displayed at both ends of the genome: U3, light gray; R, dark gray; and U5, black. The RRE-1 located in the env-coding region is indicated by the red line. The RRE-2 located in the gag-coding region overlaps with the first exon of Mat and is indicated by the green line. The six transcripts expressed by EIAV are shown at the bottom of the figure. Rev (green ellipses) binding to RRE-1 mediates nuclear export and expression of full-length transcripts encoding Gag/Pol and partially spliced transcripts encoding Env/S2. Nuclear export of transcripts encoding Tat, Rev or Grev use an endogenous cellular pathway. Rev (green ellipses) or Grev (red ellipse) binding to RRE-2 mediates nuclear export and expression of transcripts encoding Mat. Rev-mediated nuclear export of viral transcripts depends on its own multimerization (three green ellipses), whereas Grev-mediated nuclear export is not dependent on multimerization of Grev.

Export of viral mRNA from the nucleus to the cytoplasm is an important step in lentiviral replication. To date, two distinct nuclear export pathways have been identified in lentiviruses: a host-endogenous pathway used by fully spliced viral mRNAs, which is also involved in export of host mRNAs, and the Rev-CRM1 nuclear export pathway, which is utilized by unspliced or incompletely spliced transcripts (3). As a virus-encoded post-transcriptional transactivator, Rev is present in all lentiviruses, and the mechanism of Rev-mediated nuclear export of viral mRNA has been well described (3, 38, 41–43). In fact, other complex retroviruses also have post-transcriptional transactivators similar to Rev, such as Rex in human T-cell leukemia virus (HTLV) (44), Rem in mouse mammary tumor virus (MMTV) (45, 46), and Rej in Jaagsiekte sheep retrovirus (JSRV) (47). However, these retroviruses usually contain only one post-transcriptional transactivator like Rev. In this study, we have identified a novel viral protein from EIAV, in which the N-terminal part of the Gag is joined to the C-terminal part of Rev, hence the name Grev. Interestingly, Grev is found mainly in the nucleus and is able to specifically bind to viral mRNA encoding Mat to promote its nuclear export and expression, indicating that Grev functions as a post-transcriptional transactivator, similarly to Rev. Although this study does not directly address the role of Grev in EIAV pathogenicity and replication, we show that Grev is the second post-transcriptional transactivator encoded by EIAV (Fig. 8). To our knowledge, this is the first report demonstrating that two post-transcriptional transactivators are produced during the life cycle of a lentivirus.

Recently, we have identified multiple novel transcripts, including the transcript encoding Grev, in virulent strains of EIAV (LN40) and its *in vitro* adapted strains (DLV34 and DLV121) (23). These transcripts are generated by a single splicing event, but they do not share the same 5′ splice donor or 3′ splice acceptor site among themselves. However, previous studies have shown that all spliced transcripts in EIAV, including those encoding Env, Tat, Rev, and Ttm, share a 5′ splice donor site upstream of the gag-coding region (33). This result was also confirmed in our study, with the 5′ splice donor site located at nt 464 of the reference sequence (GenBank accession number GU385361.1), while the donor sites of our recently identified transcripts encoding Mat and Grev are located at nt 421 and 523 of the reference sequence. Interestingly, in an earlier study, Rosin-Arbesfeld et al. (48) isolated a rev-like transcript from EIAV-infected cells. The transcript was called p20, and it contained the second exon of Rev but lacked the first exon (48). Altogether, these results strongly suggest that EIAV alternative splicing patterns are diverse and complex. Importantly, we also confirmed that these unreported transcripts encode novel viral proteins, suggesting that the EIAV genome organization is complex and not the simplest lentivirus as previously described (22, 49).

The Grev protein is a chimeric protein that includes the first 18 amino acids of MA subunit of Gag protein and the last 82 aa of Rev. It may therefore have features or functions similar to Gag and/or Rev. Previously, Zhang et al. reported that the first 9 N-terminal amino acids of EIAV MA contain the signal that directed Gag to the TGN (50), and several studies have demonstrated that the NLS and RBD of EIAV Rev are located at the C-terminal domain of the protein (30). We observed that Grev mostly localizes in the nucleus, as does Rev. Previous studies have identified that the KRRRK motif at C-terminal of Rev is essential for its nuclear localization (30), and this result was also confirmed by the alanine substitutions in the present study. However, alanine mutation of the five basic amino acid motif KRKRK at the C terminus of Grev did not alter its nuclear localization, while Grev proteins with combinatorial mutations of this motif and its two N-terminal amino acids (DS) were retained in the cytoplasm. Therefore, the NLS of Grev may be the DSKRKRK motif at the C-terminal region rather than the KRKRK motif.

It is accepted that the major biological function of lentiviral Rev is to mediate nuclear export of unspliced and incompletely spliced transcripts by binding to RREs located within the Env-coding region (8, 19, 35, 36). Recently, we found that EIAV Rev can mediate the expression of a novel viral protein Mat through Rev specifically binding to the first exon of mat mRNA, which is located in the Gag-coding region, to promote nuclear export of mat mRNA via the CRM1 pathway (23). We therefore defined the RRE located in the Env-coding region as RRE-1, and the first exon of mat mRNA located in the Gag-coding region as RRE-2. In this study, we demonstrated that Grev can bind to RRE-2 to mediate both export of mat mRNA and the expression of Mat via the CRM1 pathway. However, Grev cannot bind to RRE-1, which is located in the Env-coding region (36) and thus cannot mediate expression of Gag/Pol. This indicates that Grev has a role as a post-transcriptional transactivator, as does Rev, but that their functions are not identical. We confirmed that the first exon of Grev is critical for its binding to mat mRNA and for Mat expression; that is, the RBD of Grev may be located in its first exon. However, there are two RBDs in Rev: an ERLE motif located in its central region and a KRRRK motif located at the C terminus, all of which could interact with different regions of the viral RNA (39). Interestingly, unlike the ^158^KRRRK^162^ motif at the C terminus of Rev as RBD, mutation of the ^94^KRKRK^98^ motif at the C terminus of Grev did not affect its binding to mat mRNA but disrupted its binding to CRM1 and its mediation of Mat expression. Thus, the ^94^KRKRK^98^ motif at the C terminus of Grev is likely to be a NES. The NES of Rev is located at the N-terminal region of its second exon, containing a number of hydrophobic residues and three leucines (31, 51). Lentiviral Rev performs nuclear export activity dependent on its own multimerization (23, 29, 37, 40). Both this and other studies (31) found that EIAV Rev, like HIV-1 Rev, also exists in multimeric forms in cells. However, we were not able to detect multimerization of Grev. Interestingly, we found that the deletion of amino acids 55 to 88 of Grev (Grev_Δ55-81_) did not affect mediation of Mat expression, but the corresponding deletion mutation in Rev rendered it inactive in mediating Mat expression (23). These results further illustrate that the mechanism by which Grev mediates nuclear export of mRNA is different from that of Rev.

It is important to know the function of a novel gene and its coding protein. However, although we have identified the novel encoded proteins Mat and Grev in EIAV, their roles in viral pathogenicity and infectivity remain unclear. We attempted to elucidate the function of Mat and Grev in counteracting the known host restriction factors and regulating viral replication under certain *in vitro* conditions, but the function remains unidentified, and we continue to work on this. However, the discovery of Grev and Mat demonstrates that the repertoire of EIAV proteins can be expanded. Such a phenomenon is not unique, and a series of novel proteins have also been identified from HIV-1 (52). This work by Imamichi et al. observed that multiple novel HIV transcripts with translationally competent ORFs were found in HIV-infected patients and demonstrated that these transcripts were produced by defective proviruses, as matching defective proviral DNA and RNA transcripts were detected from the same HIV-infected cell populations (52). However, we did not detect proviral DNA matching the grev transcript in EIAV-infected cells. Thus, it is likely that the grev transcript is produced from EIAV full-length mRNA by alternative splicing rather than from a defective provirus.

Nevertheless, a thought-provoking question remains: why does the expression of Mat use two proteins (Rev and Grev), both targeting the CRM1 pathway? Or since Rev can mediate Mat expression, why did EIAV evolve the apparently redundant Grev? One possible explanation is that Mat is critical for EIAV replication. During viral infection, the expression of viral proteins is easily disrupted by host factors. For example, it has been reported that the host restriction factor MxB can inhibit the nuclear import of HIV-1 Rev, resulting in a reduction in Rev-dependent expression of HIV-1 Gag (53). A recent study showed that the host protein Keap1 restricts Rev/RRE-dependent RNA transport, thereby inhibiting viral protein synthesis and reducing viral replication (37). From this, we speculate that Grev may be used as a “backup” for Rev to ensure expression of Mat. Alternatively, it is possible that, in addition to its effect on the expression of Mat, Grev performs other functions that are similarly important to virus replication but are dependent or independent of its nuclear export activity. Due to limited genetic content, lentiviruses encode only a few viral proteins, and each of these proteins may have many additional functions in addition to its highly conserved primary activity. The main biological function of Rev, for example, is to mediate the nuclear export of viral mRNA; however, Rev can also antagonize the antiviral activity of the host restriction factor SAMHD1 in EIAV (54). Surprisingly, one study has shown that HIV-1 Rev is able to downregulate Tat expression and viral replication via modulation of NAD(P)H:quinine oxidoreductase 1 (55). Therefore, it would be illuminating to investigate the requirements of Grev and Mat to fully understand molecular mechanisms of EIAV replication, and further work will be needed to address this question.

In summary, in this study we discovered that EIAV encodes a novel viral protein, Grev. Grev acts as a nuclear protein, like the post-transcriptional transactivator Rev, to mediate the nuclear export and expression of transcripts encoding the viral protein Mat by binding to the first exon of mat mRNAs via the CRM1 pathway. However, Grev, unlike Rev, cannot mediate EIAV Gag/Pol expression. Additionally, we also identified the NLS of Grev and found that Grev does not form multimers. These findings update the EIAV genome structure and suggest that post-transcriptional regulation patterns of EIAV are diverse and should be expanded.

## MATERIALS AND METHODS

### Antibodies and reagents.

The mouse anti-actin (A1978), mouse anti-FLAG (F1804), and mouse anti-HA (H9658) monoclonal antibodies and the rabbit anti-FLAG (F7425), rabbit anti-HA (H6908), and anti-Mouse IgG-FITC antibodies were purchased from Sigma. Mouse anti-tubulin (TA503129) monoclonal antibodies, rabbit anti-lamin (EPR8985), Alexa Fluor 568-conjugated (ab175476), and Alexa Fluor 647-conjugated (ab190565) antibodies were purchased from Abcam (Cambridge, UK). DyLight 800-labeled goat anti-mouse (5230-0415) and DyLight 680-labeled goat anti-rabbit (5230-0403) secondary antibodies were purchased from KPL. Anti-Grev and anti-p26 antibodies were prepared in our laboratory. The T7 quick high-yield RNA synthesis kit (E2050S) was purchased from NEB. The RNA 3′-end desthiobiotinylation kit (20163) and streptavidin magnetic beads (88816) were purchased from Thermo. Leptomycin B (LMB) was purchased from Beyotime Biotechnology.

### Plasmid construction and transfection.

VR1012 is a eukaryotic expression vector that contains a human cytomegalovirus (HCMV) enhancer-promoter upstream of the multiple cloning site and a bovine growth hormone polyadenylation sequence downstream of the cloning site. The Grev expression plasmid, pcDNA-Grev-HA, and VR-Grev-FLAG were constructed by inserting the *grev* gene sequence into pcDNA3.1 (+) and VR1012 vectors and then fusing HA tags or FLAG tags to the C terminus. A series of expression vectors with deletion or substitution mutations in the *grev* gene was constructed based on VR-Grev-FLAG using mutagenesis PCR. The gene sequence, which encoded amino acids 74 to 100 of Grev, was PCR amplified from the VR-Grev-FLAG and inserted into a pEGFP-C1 vector (Clontech, USA) to construct the plasmid pEGFP-Grev_74-100_. Equine CRM1 was cloned from cDNA derived from equine monocyte-derived macrophages (eMDMs) and expressed using the expression vector VR1012 as a fusion protein, with a FLAG tag at the C terminus (VR-eqCRM1-FLAG). The human CRM1 expression plasmid VR-huCRM1-FLAG (56), the codon-optimized Gag plasmids VR-Gag (50), the Mat expression plasmid pcDNA-Mat-HA, the codon-optimized Mat expression plasmid pcDNA-optMat-HA, the Mat first exon expression plasmid pcDNA-Mat-exon1-HA, the EIAV Rev expression plasmid VR-Rev-FLAG, the HIV Rev expression plasmid VR-Rev_HIV_-FLAG, and the EIAV Gag/Pol constructs pGP-RRE have been reported previously (23).

HEK293T cells and HeLa cells were cultured in Dulbecco’s modified Eagle’s medium (DMEM)-high glucose (Sigma-Aldrich, USA) supplemented with 10% fetal bovine serum (Sigma-Aldrich, USA). eMDM cells were prepared from horse whole blood as previously described (57) and cultured in RPMI 1640 (Sigma-Aldrich, USA) supplemented with 20% donor equine serum (HyClone, USA) and 40% newborn bovine serum (Ausbian, Australia). Fetal donkey dermal (FDD) cell cultures were maintained in minimal essential medium (α-MEM, Gibco, USA) supplemented with 10% fetal bovine serum (Sigma-Aldrich, USA) (57). For transfections, the corresponding plasmids were transfected using polyethyleneimine (PEI) transfection reagent (a cationic polymer, prepared by our lab) after the HEK293T cells had been grown to 80% confluence for 18 to 24 h before treatments. The cell lysates were collected 48 h after transfection.

### Amplification and identification of the grev transcript.

For amplification of EIAV transcripts, total RNAs were prepared using a TRIzol method from frozen horse tissues infected with EIAV_LN40_ (58). The tissues were homogenized in liquid nitrogen, and 1 mL of TRIzol regent was used to incubate each sample. Following precipitation of RNAs with 0.5 mL of isopropyl alcohol and washing with 1 mL of 75% ethanol, the purified total RNAs were acquired. For further identification of the grev transcript, either the EIAV virulent strain (EIAV_DLV34_) or the vaccine strain (EIAV_DLV121_), at equivalent titer was used to infect eMDM cells cultured in T25 flasks (59). At 4 days postinfection, the cells were harvested, and total RNAs were subsequently extracted by using a Bio-fast simply P RNA extraction kit (Bioer, China).

About 1 μg of RNAs were reverse transcribed into cDNAs using a PrimeScript RT reagent kit with gDNA eraser (TaKaRa, Japan) following the manufacturer’s protocols. PCR was preformed to amplify EIAV transcripts using the Kod FX Neo polymerase (Toyobo, Japan). Nested primer pairs p459 (5′-GACAGGTAAGATGGGAGACTCT-3′; forward primer)/p7685 (5′-AGTTCCTTCTTGAGCCTTAATG-3′; forward primer) and p496 (5′-CGCTCAAGAAGTTAGAGAAGG-3′; reverse primer)/p7622 (5′-TCTTTCGCTTTGAATCTCCAGG-3′; reverse primer) were used to identify grev-specific transcripts. The amplification conditions were as follows: 2 min at 98°C followed by 35 cycles of 30 s at 98°C, 30 s at 56°C and 20 s at 72°C; and finally 5 min at 72°C. PCR products were subjected to electrophoresis on 1% agarose gels, and amplified fragments were purified using a gel extraction kit (Vazyme, China) and cloned into pMD-18T vectors (TaKaRa, Japan).

To test whether the grev transcript is produced by defective provirus, total cellular DNA was extracted from eMDMs infected with EIAV_DLV121_ or EIAV_DLV34_ using the Omega blood DNA kit (Omega, USA) as a template for PCR amplification with nested primer pairs p459/p7685 and p496/p7622 to identify grev. Simultaneously, nested primer pairs p7/p8 and p7-1/p8-1 targeting the EIAV *gp90* gene were used as positive controls, and the PCR procedure was performed as described previously (60).

### RNA pulldown assay.

Biotin-modified *in vitro* RNA (Thermo scientific, USA) was dissolved in RNA structure buffer and denatured at 85°C for 5 min. Total cell proteins were extracted, incubated with 50 pmol RNA, supplemented with RIP buffer (150 mM KCl, 25 mM Tris, 5 mM EDTA, 0.5 mM dithiothreitol) to 400 μL, and then incubated at 4°C overnight. Then 50 μL streptavidin magnetic beads (Thermo scientific, USA) were washed with 1 mL binding-washing buffer (10 mM Tris-HCl [pH 7.5], 1 mM EDTA, 2 mM NaCl) for 5 min, the liquid was removed, and the beads were rinsed again. The RNA-protein conjugate and the magnetic beads were incubated with rotation at 4°C for 2 h. The magnetic beads were then washed five times with the binding-washing buffer for 5 min. Protein loading buffer was then added, and the samples were heated for the Western blotting assay.

### Western blotting.

Lysis buffer (150 mM Tris-HCl [pH 7.6], 50 mM NaCl, 5 mM EDTA, and 1% Triton X-100) was used to lyse the cells. The proteins were separated with 12% SDS-PAGE (Genscript, USA) gel electrophoresis and then transferred to NC membranes (Millipore, Germany). The membrane was placed in a blocking solution containing 5% skim milk (Biosharp, China) for 2 h at room temperature and then incubated with the indicated primary and secondary antibodies. The membrane was then analyzed on the LI-COR Odyssey imaging system (LI-COR, USA).

### Coimmunoprecipitation.

The coimmunoprecipitation experiments were performed as previously described (37). For immunoprecipitation, HEK293T cells were transfected with the indicated plasmids as described above. At 48 h post-transfection (hpt), whole-cell extracts were lysed in lysis buffer at 4°C for 30 min, and after centrifugation for 10 min at 12,000 × *g*, the supernatants were collected and incubated overnight at 4°C with anti-FLAG magnetic beads (Sigma-Aldrich, A2095). The beads were washed three to five times with ice-cold phosphate-buffered saline (PBS), and the bound proteins were eluted by mixing and heating the beads in sample loading buffer. The samples were subjected to SDS-PAGE followed by WB analysis with indicated antibodies.

### Confocal microscopy.

HeLa or FDD cells were seeded on a confocal dish and transfected with the desired vectors using PEI transfection reagent. At 36 hpt, the cells were then washed three times with PBS and fixed with 4% paraformaldehyde for 30 min, permeabilized with 0.1% Triton X-100 for 15 min, and blocked with 5% bovine serum albumin (BSA) for 1 h. To detect the intracellular distribution of Grev or Rev, the cells were incubated with anti-FLAG antibody for 2 h, washed with PBS three times, and then incubated with Alexa Flour 488- or 647-conjugated secondary antibodies for 1 h. The cell nuclei were stained with 4′,6-diamidino-2-phenylindole (DAPI), and fluorescence signals were analyzed using a confocal microscope.

### Cell fraction and quantitative real-time PCR.

HEK293T cells were seeded into 6-well plates and transfected with the desired vectors. For the preparation of nuclear and cytoplasmic RNAs, the cells were collected at 36 hpt and purified using a PARIS protein and the RNA isolation kit (Invitrogen, Thermo Fisher Scientific, USA) following the manufacturer’s instructions. An equal volume of RNAs was reverse transcribed into cDNA using the PrimeScript RT reagent kit with gDNA Eraser (TaKaRa), which has potent DNA degradation activity. To eliminate plasmid DNA contamination, all RNA samples were treated with gDNA Eraser before reverse transcription reactions. In each PCR experiment, we used one or two representative samples without adding the reverse transcription as a control to confirm that the transfected plasmid DNA had been eliminated. The copy numbers of the nuclear and cytoplasmic mRNAs from the same sample were assessed according to a previously described quantitative PCR analysis using an Aligent Mx3005P (23, 37, 56). We set the sum of nuclear and cytoplasmic mRNAs copy numbers of the same sample as 100 and evaluated the proportional distribution of the two.

### Data availability.

The *grev* sequence reported in this study was deposited to GenBank genome database under accession number ON994416.
